# Modeling of Preforming Process for Unidirectional Prepreg Composites Using Simplified Linear Friction Model and Fiber-Tracking Method

**DOI:** 10.3390/polym17101321

**Published:** 2025-05-13

**Authors:** Zhefu Li, Qinghua Song, Jun Liu, Weiping Liu, Ping Chen, Guangquan Yue

**Affiliations:** 1 Composites Center of COMAC, Shanghai Aircraft Manufacturing Co., Ltd., Shanghai 201324, China; lizhefu@comac.cc (Z.L.); songqinghua@comac.cc (Q.S.); liujun@comac.cc (J.L.); liuweiping@comac.cc (W.L.); 2 State Key Laboratory for Modification of Chemical Fibers and Polymer Materials, Center for Civil Aviation Composites, College of Materials Science and Engineering, Donghua University, Shanghai 201620, China; 3 College of Textiles, Donghua University, Shanghai 201620, China; 4 Center for Civil Aviation Composites, Donghua University, Shanghai 201620, China

**Keywords:** composite materials, finite element analysis, hot press preforming, friction model

## Abstract

Hot press preforming of unidirectional prepreg composites plays a key role in the manufacturing of aerospace components. However, defect prevention remains challenging due to complex fiber reorientation and inter-ply friction phenomena that occur during the forming process. To address these challenges, this study proposes an integrated modeling approach comprising three key components: (1) a simplified linear friction model for characterization of inter-ply slip behavior, (2) a fiber-tracking algorithm that accounts for anisotropic deformation characteristics, and (3) a coupled linear shell–membrane formulation for simultaneous modeling of in-plane and out-of-plane deformation behaviors. The proposed approach is validated through comprehensive material characterization, finite element simulation, and experimental comparisons based on a 2 m Ω-stringer geometry. Simulation results align well with experiments, showing the model’s ability to predict defects. Parametric analysis also identifies temperature as a key factor in controlling interfacial friction and improving formability, with optimal results at 75 °C. This integrated modeling approach provides an effective approach for defect prediction and process optimization, contributing to reduced material waste and improved efficiency in aerospace composite manufacturing.

## 1. Introduction

The preforming processes of unidirectional (UD) prepreg composites, particularly through diaphragm forming and hot press preforming, have become an essential manufacturing process for aerospace and automotive structural components due to its high production efficiency and excellent mechanical properties [1]. Compared to traditional manual layup techniques, these automated preforming methods, like hot press preforming, offer significant improvements in both manufacturing productivity and part consistency. Major aircraft manufacturers such as Boeing and Airbus have widely adopted hot press preforming for the fabrication of complex-shaped components, including doubly curved Ω-stringers.

However, the formation of such intricate geometries poses considerable challenges in defect prevention. During the hot press preforming of Ω-stringer structures, two primary types of defects are known to compromise structural performance: (1) wrinkling, which has been shown to reduce compressive strength [2], and (2) fiber misalignment, which leads to a decrease in laminate stiffness [3]. These defects can reduce the fatigue life of composite parts by approximately 50% [4] and tend to occur predominantly in regions of complex curvature. Accurate prediction of such defect formation is therefore essential for effective process optimization in aerospace composite manufacturing. But the accurate simulation of hot press preforming remains challenging due to several limitations in current modeling approaches.

First, the simulation must capture the evolving anisotropic behavior caused by fiber reorientation during the forming process. As prepregs undergo deformations during preforming, their mechanical properties vary accordingly due to changes in fiber orientation. Conventional methods typically neglect changes in the fiber orientation of prepreg. This leads to inaccuracies in defect prediction, particularly in regions of complex curvature where fiber realignment is most pronounced. Studies have shown that fiber angle variations can reach up to 10° in these critical regions [5,6]. To address this challenge, Peng et al. developed a non-orthogonal constitutive model for woven composite fabrics, employing a coordinate system aligned with yarn directions to simplify material characterization and accurately capture fiber reorientation during large deformations [7]. Boisse et al. analyzed and modeled three key defects in composite forming, wrinkling, fiber-induced transition zones, and large slippages of yarn, to predict and control these manufacturing flaws [8]. Thompson et al. developed a finite element model using a mutually constrained shell–membrane method to simulate severe deformations in double diaphragm forming of woven composites, successfully predicting wrinkle formation [9]. While fiber-tracking models have been proposed to improve forming simulations, most studies focus on woven prepregs, with limited attention given to UD prepregs in hot press forming processes.

The second major challenge lies in accurately modeling inter-ply behavior during prepreg preforming. Although conventional simulations often rely on Coulomb friction models to describe ply-to-ply interactions, experimental studies have revealed that prepreg friction exhibits significantly more complex characteristics under forming conditions. Sun et al. investigated the inter-ply friction mechanisms in carbon fiber/epoxy prepregs and identified a transition between Coulomb-dominated and hydrodynamic-dominated friction regimes, depending on the processing conditions [10]. To better capture such behavior, researchers have proposed more sophisticated friction laws. Dörr et al. introduced an anisotropic inter-ply friction model for finite element forming simulations, which incorporates the relative fiber orientation between adjacent plies [11]. Wang et al. investigated inter-ply slipping mechanisms during hot diaphragm preforming and proposed a three-stage analytical model for prepreg, aiming to support numerical simulation and process optimization [12]. Li et al. developed a phenomenological model that accounts for the combined effects of pressure, sliding velocity, and fiber orientation on inter-ply resistance in unidirectional prepreg [13]. However, these advanced models often suffer from high computational costs, limiting their applicability in large-scale industrial simulations.

This study aims to develop an integrated modeling approach for the hot press preforming of UD prepreg composites by integrating three key components: a temperature-dependent bilinear friction model, a real-time fiber-tracking algorithm, and a coupled shell–membrane formulation. The primary objective is to address critical challenges in defect prediction and process optimization that arise during the formation of complex composite structures. First, a bilinear friction model is introduced, which captures the nonlinear stress-slip response while maintaining computational efficiency suitable for simulations. Second, a real-time fiber-tracking approach is implemented to dynamically update the anisotropic properties of UD prepregs during deformation. Third, a coupled shell–membrane method is incorporated into the integrated modeling approach to enable precise characterization of out-of-plane deformation responses in UD prepreg materials. The proposed modeling approach is rigorously validated through comprehensive experimental investigations on Ω-stringer components, demonstrating its accuracy and practical applicability in aerospace manufacturing.

## 2. Method

**Figure 1:**
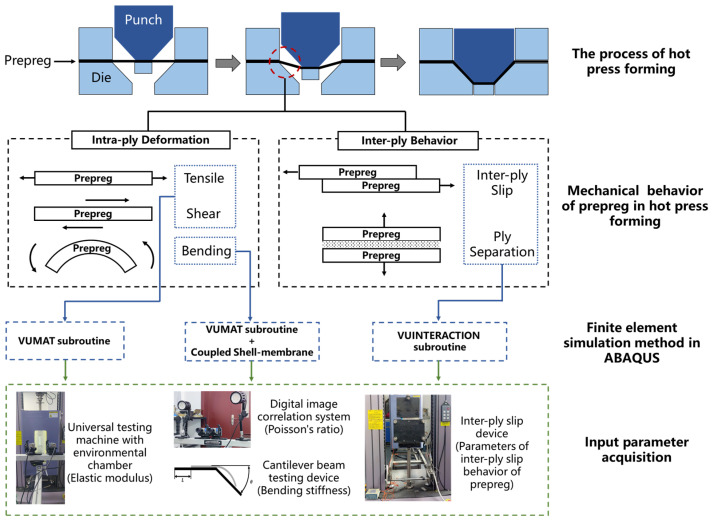
Deformation behavior of prepreg during hot press forming and finite element modeling framework.

During hot press preforming, prepregs predominantly exhibit two categories of mechanical behavior: intra-ply deformation and inter-ply behavior (Figure 1). Intra-ply deformation includes tensile, shear, and bending responses within individual plies, whereas inter-ply behavior mainly involves inter-ply friction and ply separation. Accordingly, the finite element model of prepregs will be developed to accurately simulate these mechanical responses in the numerical simulation.

### 2.1. Intra-Ply Deformation

The simulation process of the hot press preform was conducted using the Abaqus finite element software. In Abaqus/Explicit (Abaqus/CAE 2018), the VUMAT subroutine is employed to characterize the intra-ply behavior. Unlike isotropic materials, prepregs exhibit pronounced anisotropy due to directional differences in their mechanical properties. In UD prepreg, the fibers are aligned parallel to each other, resulting in significantly higher stiffness and strength along the fiber direction compared to the transverse direction. During the preforming process, prepregs undergo deformation that induces fiber reorientation, resulting in corresponding variations in their mechanical properties. However, conventional built-in material models in Abaqus—such as the Laminate model—are unable to dynamically update fiber orientations during forming simulations. This key limitation leads to inaccurate predictions of orientation-dependent property changes, particularly in unidirectional prepregs, where fiber reorientation influences the mechanical behavior. To address this limitation, the VUMAT subroutine is implemented to dynamically update the material properties based on real-time fiber orientation.

In this study, following the modeling approach of tracking fiber orientation and drawing inspiration from the preferred fiber orientation model and HypoDrape model, a model was developed to describe the deformation behavior of prepreg during the thermoforming preforming process [9,14,15,16,17]. Based on the constitutive relationship of hypoelastic materials, the computational methodology is outlined as follows:

(1) $$\begin{array}{c} \sigma^{▽}=C:D \end{array}$$

where $\sigma^{▽}$ represents the objective stress rate, D denotes the strain rate tensor, and C is the material constitutive tensor defined by fiber orientation. In the explicit solution process of the Abaqus/VUMAT subroutine, the Green–Naghdi stress rate is primarily employed. This objective stress rate eliminates the influence of rigid-body rotations on the stress evolution, which can be expressed as follows:

(2) $$\begin{array}{c} \sigma^{▽G}=\frac{D\sigma }{Dt}-\Omega \cdot \sigma -\sigma \cdot \Omega^{T} \end{array}$$

where Ω represents the angular velocity tensor associated with rigid-body rotation.

From Equation (2), it follows that accurate large-deformation analysis using the Green–Naghdi stress rate requires correction of the rotational effects on the Cauchy stress rate induced by rigid-body motion. Abaqus/Explicit employs a polar rotation-based correction method [18]:

(3) $$\begin{array}{c} g_{i}=R\cdot g_{i}^{0} \end{array}$$

Here, $g_{i}$ denotes the material-rotating local orthogonal coordinate system defined in Abaqus, commonly referred to as the Green–Naghdi frame; $g_{i}^{0}$ denotes the initial Green–Naghdi coordinate system; subscript i indicates different axes of the coordinate system; and R is the rotation tensor. The rotation tensor can be obtained through polar decomposition [18]:

(4) $$\begin{array}{c} R=F\cdot U^{-1} \end{array}$$

where F is the deformation gradient tensor and **U** represents the right stretch tensor. The orientation evolution of fibers and fibers’ normal direction in prepreg (the detailed definition of prepreg orientation is illustrated in Figure S1) can be described as follows [9]:

(5) $$\begin{array}{c} f_{i}=\frac{F\cdot f_{i}^{0}}{\left\|F\cdot f_{i}^{0}\right\|}=\frac{F\cdot g_{i}^{0}}{\left\|F\cdot g_{i}^{0}\right\|} \end{array}$$

where $f_{i}$ represents the updated orientation matrix for both the fiber direction and its transverse normal direction. Initially, the fibers are aligned with the Green–Naghdi coordinate axes. The relationship between fiber orientation and Green–Naghdi axes during deformation can be characterized by the transformation matrix $T_{i}$ [9]:

(6) $$\begin{array}{c} T_{i}=\left[\begin{array}{cc} cos\alpha_{i} & -sin\alpha_{i}\\ sin\alpha_{i} & cos\alpha_{i} \end{array}\right] \end{array}$$

So, the strain increments in Green–Naghdi coordinates can be transformed to the fiber coordinate system through the following [9]:

(7) $$\begin{array}{c} d\varepsilon_{f_{i}}={T_{i}}^{T}d\varepsilon T_{i} \end{array}$$

Subsequently, stress increments in the fiber-aligned directions are computed using the prepreg’s stiffness matrix ($C_{i}$) [9]:

(8) $$\begin{array}{c} d\sigma_{f_{i}}=C_{i}d\varepsilon_{f_{i}} \end{array}$$

Finally, the computed stress increments are transformed back to Green–Naghdi coordinates via a matrix $T_{i}$ to complete the incremental step. The fiber tracking method implemented in the Abaqus VUMAT subroutine enables effective modeling of prepreg layers during thermoforming processes. The fiber rotation angles can be obtained by outputting parameters from the transformation matrix $T_{i}$ in the VUMAT subroutine. As indicated from Equation (8), the essential material parameters to be characterized in the VUMAT subroutine include the tensile modulus of the prepreg, Poisson’s ratio, shear modulus, and fiber orientation angle.

In comparison to its length and width, the thickness of unidirectional prepreg is relatively small. Therefore, many researchers use membrane elements without bending stiffness to simulate the deformation of prepreg [19,20,21]. However, recent studies have shown that incorporating bending stiffness into the model can significantly improve simulation accuracy, particularly in predicting defects such as wrinkling and buckling. In Abaqus, membrane elements are parts with in-plane strength but no bending stiffness, while shell elements possess both in-plane strength and bending stiffness. The bending stiffness of shell elements in Abaqus cannot be directly defined and must be calculated from the assigned material properties. If shell elements alone are used to simulate single-layer prepreg, the high elastic modulus in the fiber direction would result in an excessively large bending modulus [9].

To overcome the limitations of using membrane elements and shell elements separately, this investigation has used a “coupled shell” element. The deformation behavior of single-layer prepreg is decoupled into in-plane mechanical behavior and out-of-plane mechanical behavior [9,22]. The membrane element is responsible for simulating the in-plane mechanical behavior of the prepreg, while the shell element is responsible for simulating the out-of-plane bending behavior. The nodes and elements of the two types of elements are merged, allowing them to constrain each other and simulate the deformation behavior of the prepreg. The in-plane constitutive behavior is defined through the VUMAT subroutine, while the shear stiffness of the shell element is set to zero, ensuring that in-plane shear deformation is governed exclusively by the membrane component. The elastic modulus of the shell element is determined by Equation (9) [9]:

(9) $$\begin{array}{c} G=\frac{E{h_{p}}^{3}}{12} \end{array}$$

where G is the bending stiffness, E is the equivalent tensile modulus of the shell element, and $h_{p}$ is the thickness of the single-layer prepreg.

### 2.2. Inter-Ply Behavior

In this study, the VUINTERACTION subroutine was utilized to model the inter-ply interactions. The penalty method was employed to simulate the inter-ply separation at the interfaces between prepregs [23]. In the corresponding subroutine, the contact state of the surfaces is characterized by the sign of the distance of the node. Specifically, a negative distance of node indicates that the two surfaces are in a separated state, while a positive value implies mutual penetration between the surfaces. When the surfaces are detached, an outward-directed cohesive force is applied to represent the inherent inter-ply adhesion mechanism present in uncured prepregs. This force accounts for the bonding forces that exist between adjacent prepreg layers under non-contact conditions. Conversely, when penetration occurs, an inward-directed repulsive force is imposed to prevent excessive interpenetration.

Consequently, the inter-ply behavior between prepregs can be described by the following equation:

(10) $$\begin{array}{c} \left\{\begin{array}{c} \sigma_{N}=10000^{\left|u\right|};\;\;u\leq 0\\ \sigma_{N}=v\cdot u;\;\;u>0,\;\sigma_{N}\geq \sigma_{max}\\ \sigma_{N}=0;\;\;u>0,\;\sigma_{N}\geq \sigma_{max} \end{array}\right. \end{array}$$

When u≤0, a penalty function is applied to effectively prevent interpenetration between contacting surfaces [24]. In this formulation, v and $\sigma_{max}$ represent the bonding strength coefficient and the maximum bonding stress between the prepregs, respectively. The interfacial adhesion behavior between prepreg plies is primarily governed by the resin matrix, where the bonding strength coefficient and the maximum bonding stress represent the adhesive characteristics of the resin system. Given that the mechanical properties in the transverse (fiber-normal) direction of the prepreg are also dominated by the resin phase, these interfacial parameters can be experimentally determined through mechanical testing of the prepreg.

During the forming process, inter-ply slip exhibits three distinct mechanical response regimes governed by the magnitude of slip displacement: (1) an initial linear elastic stage characterized by a proportional stress-slip relationship, (2) an intermediate yield stage exhibiting logarithmic stress-slip behavior, and (3) a final hardening stage demonstrating renewed linear stress-slip correlation [25]. Although the yield regime represents a transitional phase within the overall interlaminar slip response, it can be reasonably approximated using a simplified bilinear model. This approach significantly improves computational efficiency while retaining the essential mechanical characteristics. This modeling approach captures the essential mechanical behavior by combining the initial stiffness ($k_{1}$) and hardening stiffness ($k_{2}$) responses while eliminating the nonlinear transitional complexity. The constitutive representation of inter-ply slip resistance can be expressed as follows:

(11) $$\begin{array}{c} \left\{\begin{array}{c} \sigma_{T}=\sigma_{T}^{*}+k_{1}∆L;\;\;u\leq 0,\sigma_{T}\leq \sigma_{y}\\ \;\sigma_{T}=\sigma_{T}^{*}+k_{2}∆L;\;\;u\leq 0,\sigma_{T}>\sigma_{y}\;\\ \sigma_{T}=0;\;\;u>0 \end{array}\right. \end{array}$$

where $\sigma_{T}$ denotes the inter-ply slip resistance. When u ≤ 0, it indicates compressive contact between plies. When u > 0, it signifies interfacial separation, with no tangential traction transmitted across the interface. $\sigma_{T}^{*}$ represents the nodal stress value before updating. $\sigma_{y}$ denotes the critical inter-ply shear stress at the linear-to-hardening transition point. ∆L refers to the relative slip displacement, obtained from the data of the Abaqus VUINTERACTION Subroutine.

## 3. Experiments

### 3.1. Materials

The M21/UD194/IMA unidirectional prepreg (Hexcel Co., Stamford, CT, USA) was employed in the experimental study. The thickness of the prepreg ply is 0.187 mm. 

### 3.2. Inte-Ply Slip Testing

**Figure 2:**
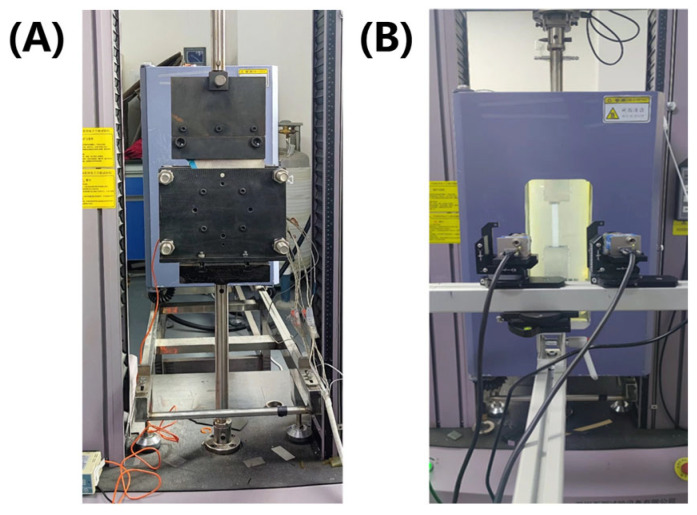
(**A**) Inter-ply slip measurement device for prepreg; (**B**) Universal testing machine with environmental chamber and DIC system.

As illustrated in Figure 2A, the inter-ply slip behavior of the unidirectional prepreg composite was quantitatively evaluated using a customized interfacial slip testing apparatus developed in our previous study [13]. In this work, two representative temperatures of 40 °C and 75 °C were selected for experimental investigation based on the temperature-dependent viscosity characteristics of the prepreg (Figure S2). The viscosity test results revealed that at 40 °C, the prepreg exhibited significantly higher viscosity, which was consequently adopted in finite element simulations to represent conditions with greater inter-ply slip resistance. Conversely, the prepreg viscosity reached a stable low-viscosity plateau at 75 °C, making this temperature suitable for simulating scenarios with reduced inter-ply slip resistance in the numerical models.

### 3.3. Mechanical Properties Testing

The mechanical properties of the prepreg are critical input parameters for finite element simulations. In accordance with ASTM D3039-00 standard [26], the tensile properties of unidirectional prepregs were characterized in both the fiber direction and the fiber’s normal direction at 40 °C and 75 °C. The specimen dimensions were 180 mm × 15 mm. For the tensile property experiments in the fiber direction, the prepreg samples consisted of a single ply. For tensile testing in the fiber direction, single-ply specimens were used. However, due to the lack of an established test standard for characterizing the transverse mechanical properties of unidirectional prepregs, a non-standardized stacked-ply configuration was adopted [27]. In this study, the tensile specimens of fiber normal direction consisted of ten plies stacked and vacuum-compacted at room temperature for 1 h prior to testing.

Owing to resin softening at elevated temperatures, conventional strain gauges could not be reliably attached to the prepreg surface. Therefore, a Digital Image Correlation (DIC, VIC-3D SYSTEM, Correlated Solutions INC, Irmo, SC, USA) system was employed to measure material strain. As shown in Figure 2B, the experimental setup included a universal testing machine (TM 204C, Wance Testing Machine Co., Ltd., Shenzhen, China) equipped with an environmental chamber and the DIC system, enabling simultaneous measurement of longitudinal and transverse strains for accurate determination of Poisson’s ratio. All tests were performed at 40 °C and 75 °C, with three replicates conducted under each condition. The results were averaged to ensure statistical reliability and consistency in material characterization.

### 3.4. Flexural Stiffness Testing

**Figure 3:**
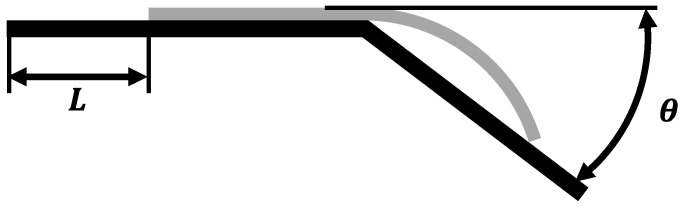
Test principle of flexural stiffness cantilever beam.

In accordance with the ISO 9073-7 standard [28], a cantilever beam setup was employed to characterize the bending stiffness of unidirectional prepregs in both the fiber direction and the fiber’s normal direction. Figure 3 illustrates the schematic diagram of the testing principle for bending properties. During testing, the specimen moves uniformly to the right and deflects under its own weight. When the deflection angle θ reaches 41.5°, the movement distance L is recorded, and the bending stiffness is calculated. The testing temperatures were set at 40 °C and 75 °C.

### 3.5. Hot Press Preforming and Finite Element Analysis

**Figure 4:**
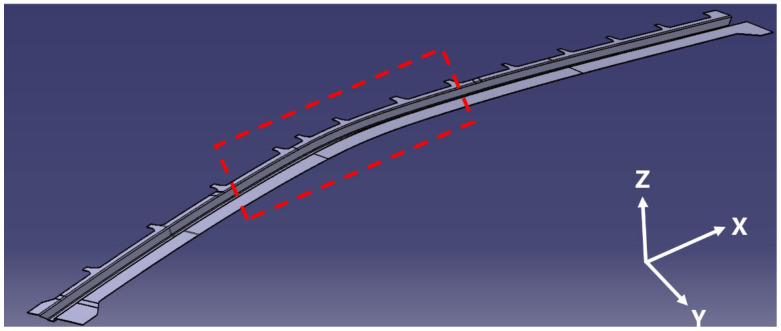
Double curved Ω-stringer in hot press preforming. (Target Ω-stringer geometry in CAE model highlighted by red dashed box).

The red-framed region in Figure 4 indicates the target geometry: a 2 m doubly-curved Ω-stringer structure with complex deflections in both X-Z and Y-Z planes.

**Figure 5:**
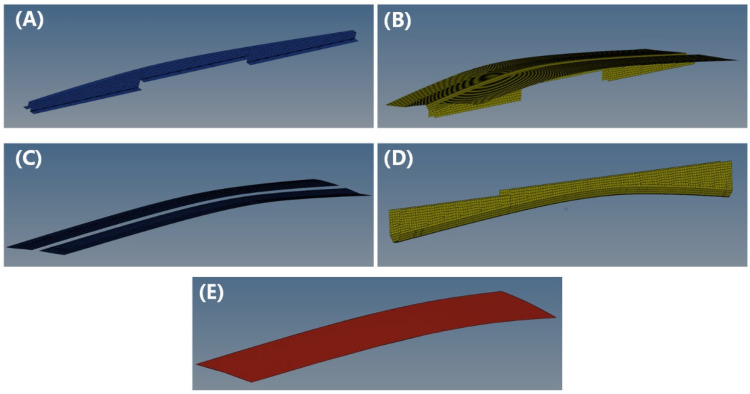
Mold and prepreg digital model processed by HyperMesh software (**A**) Core; (**B**) Die; (**C**) Blank holder; (**D**) Punch; (**E**) Prepreg.

The simulation model for hot press preforming of doubly-curved Ω-stringers was constructed through the following steps: (1) Mold geometry modeling using CATIA (CATIA V5R18); (2) Finite element meshing in HyperMesh (HyperMesh 2021). The assembly consists of four functional parts: core, die, blank holder, punch, and prepreg; (3) Computational optimization of the model, which involved selective surface extraction (retaining only prepreg-contact surfaces) and geometric repair. Figure 5 presents the finalized finite element model after preprocessing.

**Figure 6:**
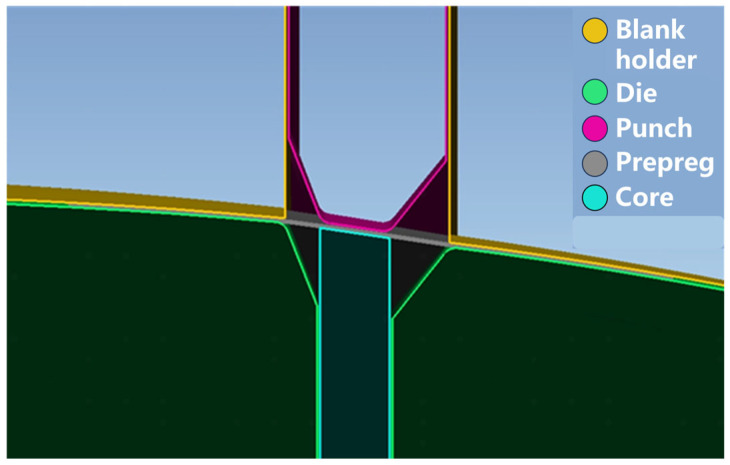
Schematic diagram of CAE simulation model of double curved Ω-stringer.

The individual part models were sequentially imported into Abaqus for final assembly. Figure 6 presents the sectional view of the complete hot-press preforming simulation assembly. The prepreg was represented through a coupled shell- membrane approach combining membrane elements (M3D4) and shell elements (S4R). The prepreg layup followed a stacking sequence of [45/0_2_/−45/90]_s_. The prepreg was modeled in a free state, with deformation driven solely by contact interactions with the tooling components.

During hot press preforming, wrinkles frequently develop in prepregs under compressive loading. Wrinkling in composite materials fundamentally originates from the bending of prepreg, which is manifested as localized rotations of elements in simulations [29]. These characteristic rotations are quantitatively captured through UR values. Specifically, significant deviations in UR values within regions expected to undergo minimal rotation, such as the crown transition zones of Ω-stringer components, serve as clear evidence of wrinkle initiation and development.

## 4. Results and Discussion

**Figure 7:**
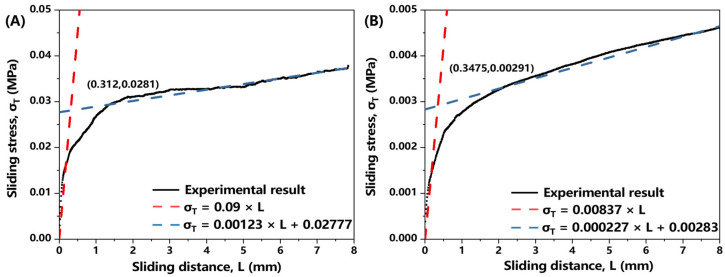
The experimental results of inter-ply slip behavior: (**A**) 40 °C; (**B**) 75 °C.

Figure 7 presents the experimental results of inter-ply slip behavior, demonstrating that the proposed simplified linear friction model accurately captures the measured friction characteristics of the prepreg. The linear formulation effectively represents both the initial elastic response and the subsequent hardening behavior. Table 1 summarizes the parameters for Equation (11) at both investigated temperatures.

**Figure 8:**
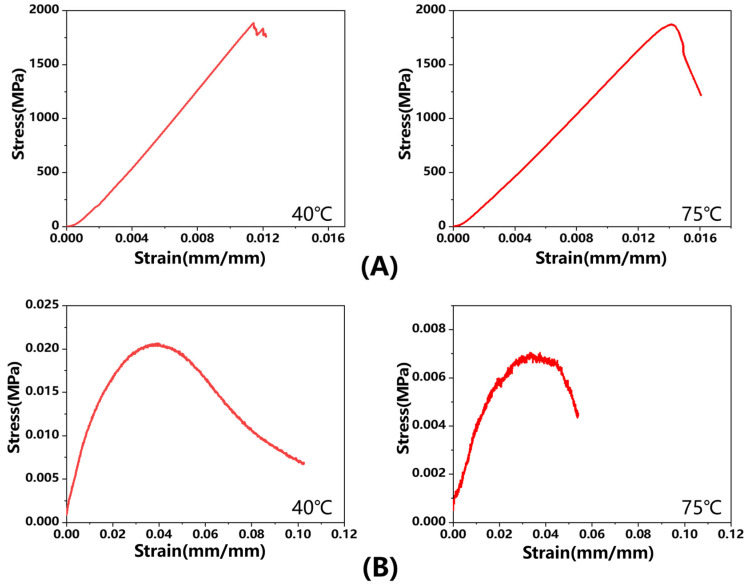
(**A**) Tensile curve in fiber direction at 40 °C and 75 °C; (**B**) Tensile curve in fiber’s normal direction at 40 °C and 75 °C.

The experimental results of tensile tests conducted at various temperatures are presented in Figure 8. As shown in Figure 8A, temperature has a negligible effect on the tensile properties in the fiber direction. This phenomenon can be attributed to the stable mechanical performance of carbon fibers within the temperature range of 40–75 °C. Figure 8B presents the tensile test results of the fiber’s normal direction in prepreg at different temperatures. The results showed a progressive decrease in tensile strength with increasing temperature, which is primarily governed by the resin-dominated deformation mechanism. Elevated temperatures reduce resin viscosity and weaken interfacial bonding, leading to a corresponding decline in tensile modulus. Table 2 summarizes the experimental data on tensile properties.

The shear modulus of the prepreg is determined by Equation (12):

(12) $$\begin{array}{c} G_{12}=\frac{E_{2}}{2\left(1+\upsilon_{12}\right)} \end{array}$$

where G_12_ represents the in-plane shear modulus, E_2_ denotes the elastic modulus in the fiber’s normal direction, and υ_12_ is the Poisson’s ratio. The calculated shear modulus values are summarized in Table 3. The results demonstrate a progressive decrease in shear modulus with increasing temperature. This trend can be attributed to the temperature-induced decrease in resin viscosity, which significantly weakens the prepreg’s resistance to shear deformation.

The bending properties of prepregs were tested along different material orientations at various temperatures. The bending stiffness and equivalent tensile modulus were calculated using Equation (9), with results presented in Table 4.

**Figure 9:**
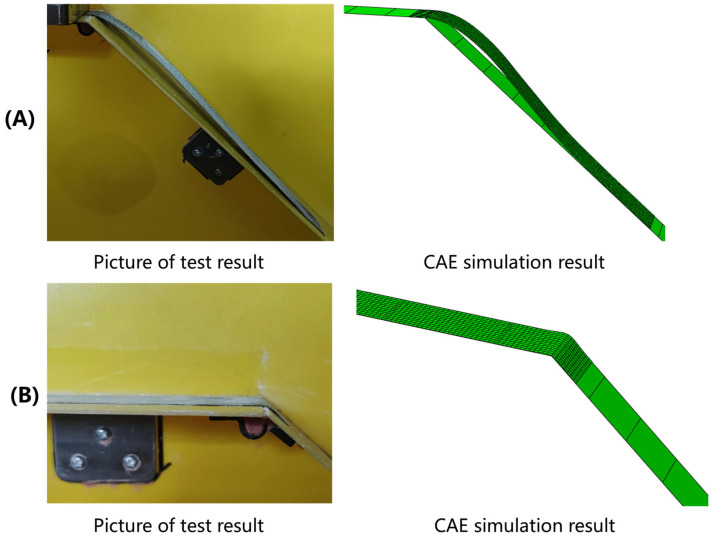
(**A**) Bending stiffness test result and simulation result in the fiber direction; (**B**) Bending stiffness test result and simulation result in the fiber’s normal direction.

Figure 9 demonstrates that the coupled shell-element formulation successfully captures the anisotropic out-of-plane bending behavior observed experimentally in both the fiber direction and the fiber’s normal direction. The simulation results exhibit close agreement with experimental measurements, as indicated by root mean square error (RMSE) values of 1.27 mm (the fiber direction, 40 °C) and 0.91 mm (the fiber direction, 75 °C), with corresponding errors of 0.25 mm and 0.21 mm in the fiber’s normal direction. These small deviations are primarily attributed to minor variations in prepreg areal density. The strong correlation between simulated and experimental results validates the capability of this method to capture direction-dependent bending stiffness with high fidelity.

**Figure 10:**
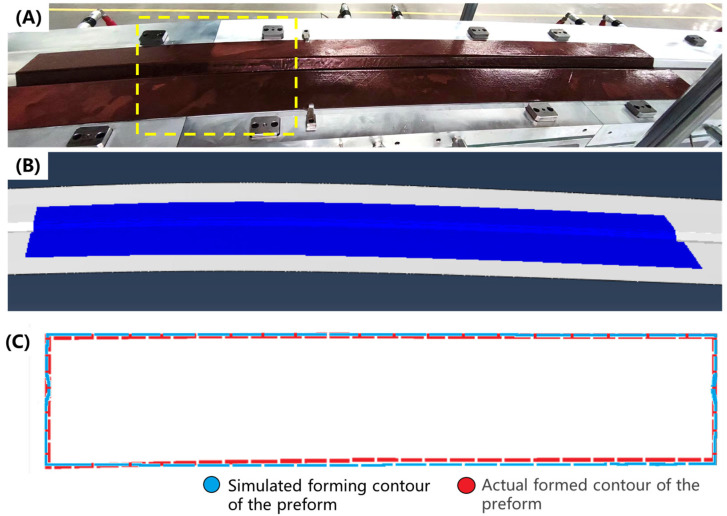
Preform of 2 m double curved Ω-stringer: (**A**) Picture of sample; (**B**) CAE simulation result; (**C**) Preform contour comparison with the result of finite element simulation. (The crown zone of the Ω-stringer geometry highlighted by yellow dashed box).

As shown in Figure 10A,B, the finite element simulation closely replicates the experimentally observed geometry of the prepreg after forming at 75 °C. In order to characterize the accuracy of the simulation, select reference lines on the die served as benchmarks to record the distance between the deformed prepreg edge and these reference lines at multiple points (Figure S3). These measurements were then reconstructed to compare with the simulated contour from Abaqus. Figure 10C provides a direct overlay comparison between simulated and experimental contours. The deviations were quantified using RMSE, which showed an accuracy of 1.27 mm. This outcome confirms the accuracy of the proposed modeling method during hot press preforming.

**Figure 11:**
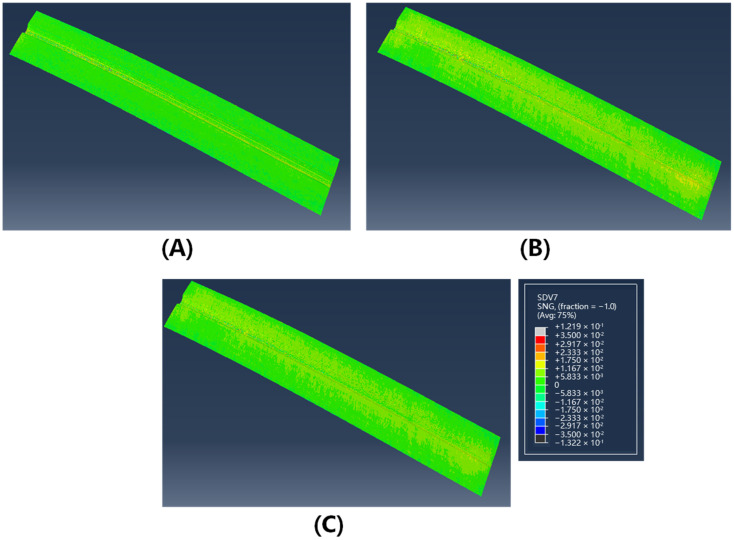
The sin value of fiber deflection angle of double curved Ω-stringer at 40 °C: (**A**) 0° layer; (**B**) 45° layer; (**C**) 90° layer.

**Figure 12:**
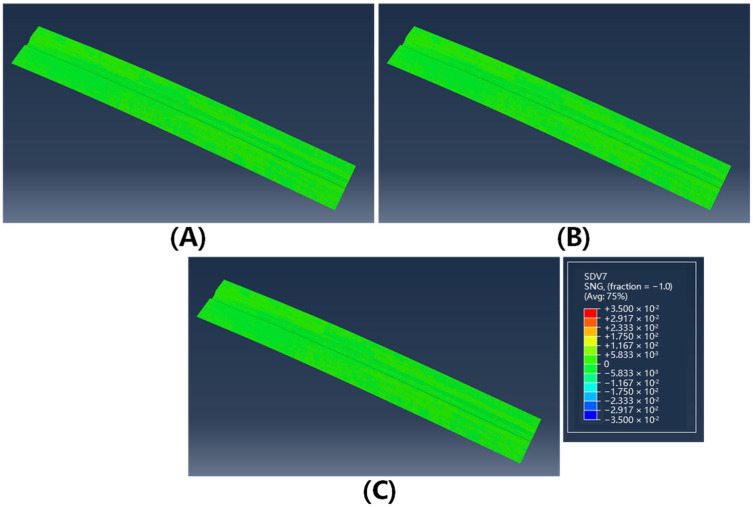
The sin value of fiber deflection angle of double curved Ω-stringer at 75 °C: (**A**) 0° layer; (**B**) 45° layer; (**C**) 90° layer.

In Figure 11 and Figure 12, the contour plots of sine values for fiber orientation angles in the formed structures at 40 °C and 75 °C are presented. The results demonstrate a significant reduction in fiber misalignment with increasing forming temperature, where the maximum fiber deviation decreases from 7.6° at 40 °C to less than 2° at 75 °C. This temperature-dependent behavior can be attributed to enhanced inter-ply slip behavior at elevated temperatures, which effectively reduces shear coupling between plies and consequently mitigates fiber angle deviation. Spatial distribution analysis indicates that fiber angle deviation is most pronounced in the crown zone of the Ω-stringer geometry—the region highlighted by the yellow box in Figure 10A—where the curvature undergoes the most abrupt variation.

**Figure 13:**
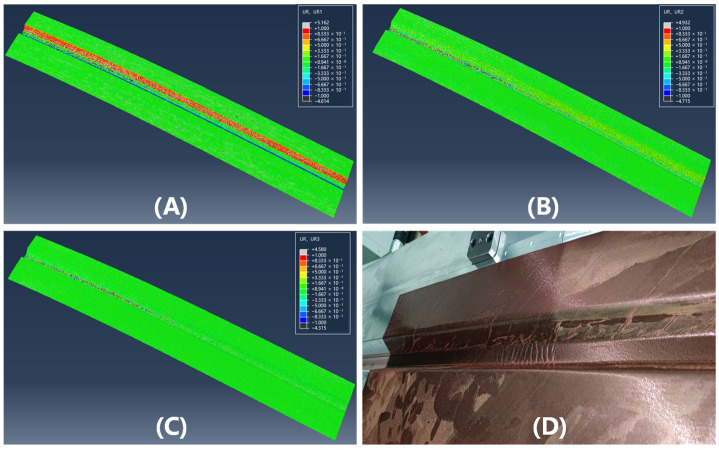
Nephograms of surface rotating parts of double curved Ω-stringer and the sample picture at 40 °C: (**A**) UR1; (**B**) UR2; (**C**) UR3; (**D**) sample picture.

**Figure 14:**
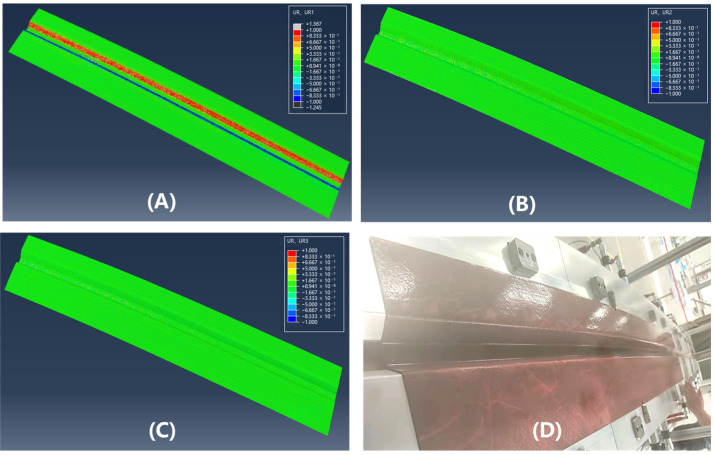
Nephograms of surface rotating parts of double curved Ω-stringer and the sample picture at 75 °C: (**A**) UR1; (**B**) UR2; (**C**) UR3; (**D**) sample picture.

Figure 13 and Figure 14 illustrate the simulation of wrinkles and corresponding experimental photographs of the formed parts at 40 °C and 75 °C, respectively. To systematically evaluate wrinkle formation, the L/D ratio (wrinkle length-to-height) was employed as a key evaluation metric, where wrinkles are classified as defective under the criterion that L/D < 200. Through geometric analysis of wrinkle morphology and ply rotational behavior in finite element models, a corresponding threshold for the rotational degree (UR) was defined at UR = 0.01, which indicates the non-green areas in Figure 13 and Figure 14. Consistent with the fiber orientation deviation results, the magnitude of surface rotation parts decreases with increasing forming temperature, indicating that elevated temperatures can effectively suppress wrinkle formation. This phenomenon can be attributed to the reduced inter-ply slip resistance at higher temperatures, which lowers the stresses acting on the plies and thereby mitigates their tendency to wrinkle. The region most susceptible to wrinkling defects is concentrated in the crown area of the geometry, which aligns well with experimental observations. This spatial correlation demonstrates that the crown region of doubly-curved structures exhibits the highest probability of surface wrinkling defects, a tendency that can be effectively controlled through optimization of the forming temperature parameter.

**Figure 15:**
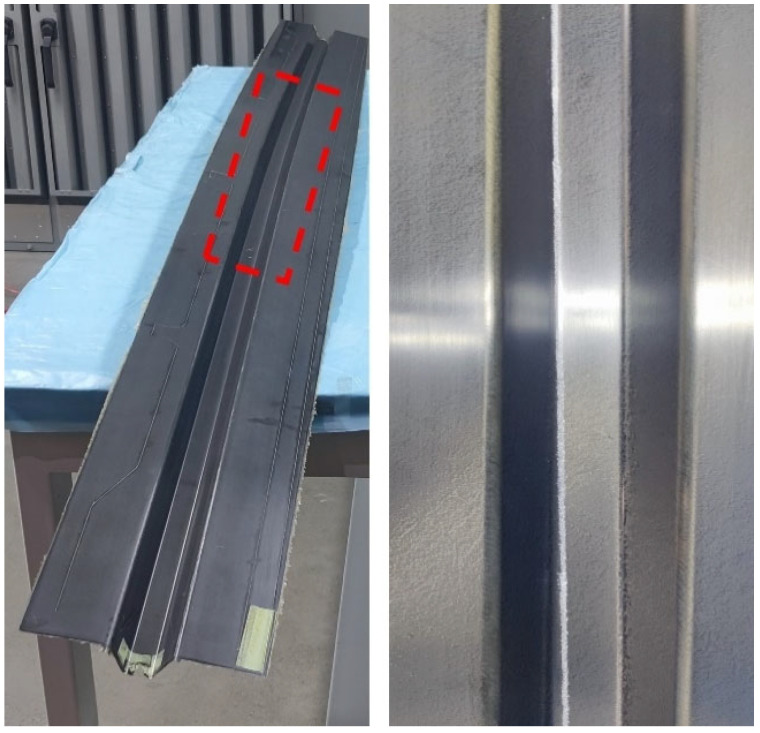
A 2 m double curved Ω-stringer after curing. (The crown zone of the Ω-stringer geometry highlighted by red dashed box).

The analysis demonstrates that optimal part quality is achieved at 75 °C, which minimizes both fiber reorientation and surface wrinkling. Figure 15 presents the surface morphology of the 2 m doubly curved stringer after autoclave curing, fabricated using the optimized forming temperature. The red box highlights the critical region identified in finite element simulations as being prone to defect formation. As shown in the figure, the manufactured component exhibits excellent surface quality with no visible wrinkling defects. This observation confirms the predictive accuracy of the simulation method in capturing defect formation in composite structures with complex curvature.

## 5. Conclusions

This study presents an integrated modeling approach for simulating the hot press preforming of UD prepreg composites, effectively addressing key challenges in defect prediction and process optimization.

(1) This study elucidates the fundamental mechanisms governing the behavior of UD prepregs during hot press preforming by explicitly characterizing intra-ply deformations (in-plane tensile/shear and out-of-plane bending) and inter-ply interactions (slip and separation). It designs dedicated experimental methods to quantify these behaviors, including tensile testing, cantilever beam bending, and custom interfacial slip tests, and integrates the resulting insights into an integrated modeling approach that couples material anisotropy, friction dynamics, and deformation;
(2) A simplified bilinear friction model was employed to capture inter-ply slip behavior under varying temperatures. Experimental validation confirmed that elevated temperatures (75 °C) reduce inter-ply friction and improve formability. The model showed good agreement with experimental data (RMSE < 9.5 × 10^−7^ MPa), demonstrating its capability in accurately simulating the frictional behavior of UD prepreg materials;
(3) A fiber-tracking method was applied for UD prepreg in the process of hot press preform by updating material properties in real-time based on fiber reorientation. By decoupling in-plane (membrane elements) and out-of-plane (shell elements) behaviors, the model accurately predicts bending stiffness;
(4) Experimental validation using a 2 m doubly curved Ω-stringer component confirmed the accuracy of the proposed integrated modeling approach. A comparison of the surface contour between simulation results and experimental measurements yielded a root mean square error (RMSE) of 1.27 mm, indicating good agreement. Elevated processing temperatures (75 °C) demonstrated significantly improved wrinkle suppression compared to conventional forming at 40 °C, consistent with simulation predictions.

The integrated modeling approach provides a practical tool for process optimization in hot press preform, enabling defect prevention and quality control for complex geometries. Future work should focus on extending the friction model for more complex interfacial behaviors and incorporating additional material characterization data.

## Figures and Tables

**Table 1:** Parameters of inter-ply slip behavior at 40 °C and 75 °C.

Temperature (°C)	Initial Stiffness k_1_ (MPa/mm)	$\mathrm{Yield}\;\mathrm{Stress}\;\sigma_{y}$ (MPa)	Hardening Stiffness k_2_ (MPa/mm)	RMSE
40	0.09	0.0281	0.00123	9.45 × 10^−7^
75	0.00837	0.00291	0.000227	8.49 × 10^−7^

**Table 2:** Tensile test results in the fiber direction and the fiber’s normal direction.

Temperature (°C)	Sample Direction	Tensile Modulus (GPa)	Poisson’s Ratio
40	fiber direction	141.98	0.3731
75	fiber direction	141.62	0.3775
40	fiber’s normal direction	0.815	0.0029
75	fiber’s normal direction	0.562	0.0035

**Table 3:** Shear modulus of prepreg.

**Temperature (°C)**	**Shear Modulus (MPa)**
40	0.2963
75	0.2046

**Table 4:** Bending stiffness and equivalent tensile modulus.

Temperature (°C)	40	75
Density (g/m^2^)	294	294
Displacement of fiber direction sample (cm)	26.9	19.1
Displacement of fiber’s normal direction sample (cm)	2.0	1.6
Bending stiffness in fiber direction (mN·cm)	5722.7	2048.6
Bending stiffness in fiber’s normal direction (mN·cm)	2.4	1.2
Equivalent tensile modulus in fiber direction (MPa)	1177.5	421.5
Equivalent tensile modulus in fiber’s normal direction (MPa)	0.5	0.3

## Data Availability

The original contributions presented in this study are included in the article/Supplementary Material. Further inquiries can be directed to the corresponding authors.

## Supplementary Materials

The following supporting information can be downloaded at: https://www.mdpi.com/article/10.3390/polym17101321/s1, Figure S1: Terminology for UD prepreg fiber orientation definition. Figure S2: Viscosity—temperature curve of prepreg. Figure S3: The schematic diagram of reference lines on the die.
