# Supplementary material for: Modeling of Preforming Process for Unidirectional Prepreg Composites Using Simplified Linear Friction Model and Fiber-Tracking Method
Source: Polymers (Basel). 2025 May 13;17(10):1321. doi: 10.3390/polym17101321 (PMC12114742; doi:10.3390/polym17101321)
Supplement: Supplementary file 1 [file polymers-17-01321-s001.zip › polymers-3609796-supplementary.pdf]

Modeling of Preforming Process for Unidirectional Prepreg Composites Using Simplified Linear Friction Model and Fiber-Tracking Method

Zhefu Li [1], Qinghua Song [1], Jun Liu [1,2], Weiping Liu [1,2], Ping Chen [1,*] and Guangquan Yue [3,4,*]

[1] Composites Center of COMAC, Shanghai Aircraft Manufacturing Co., Ltd., Shanghai 201324, China; lizhefu@comac.cc (Z.L.); songqinghua@comac.cc (Q.S.); liujun@comac.cc (J.L.); liuweiping@comac.cc (W.L.)
[2] State Key Laboratory for Modification of Chemical Fibers and Polymer Materials, Center for Civil Aviation Composites, College of Materials Science and Engineering, Donghua University, Shanghai 201620, China
[3] College of Textiles, Donghua University, Shanghai 201620, China
[4] Center for Civil Aviation Composites, Donghua University, Shanghai 201620, China
* Correspondence: chenping@comac.cc (P.C.); yueguangquan@dhu.edu.cn (G.Y.)

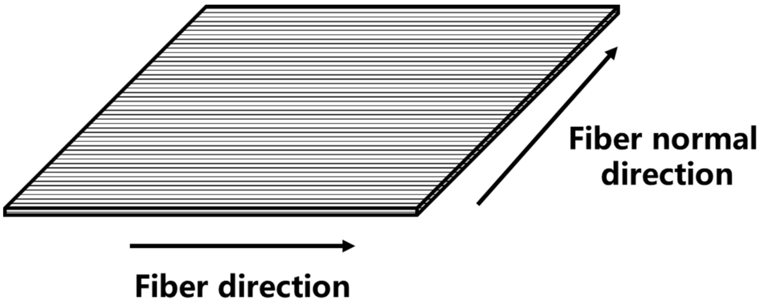

Figure S1 Terminology for UD prepreg fiber orientation definition

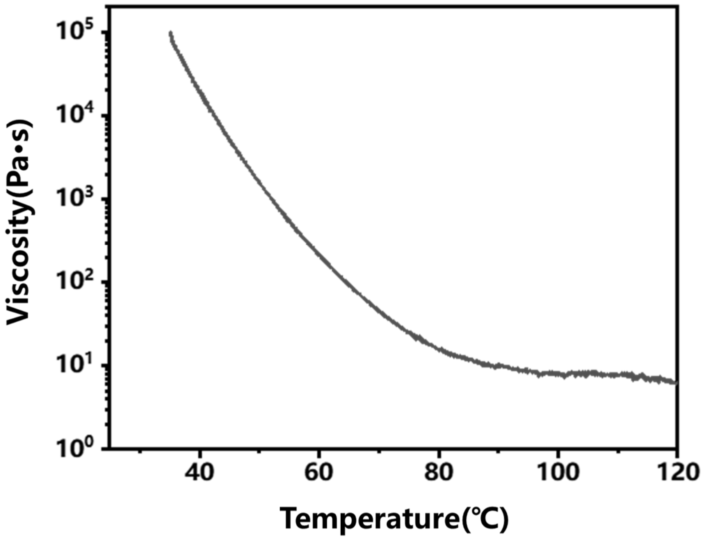

Figure S2 Viscosity - temperature curve of prepreg

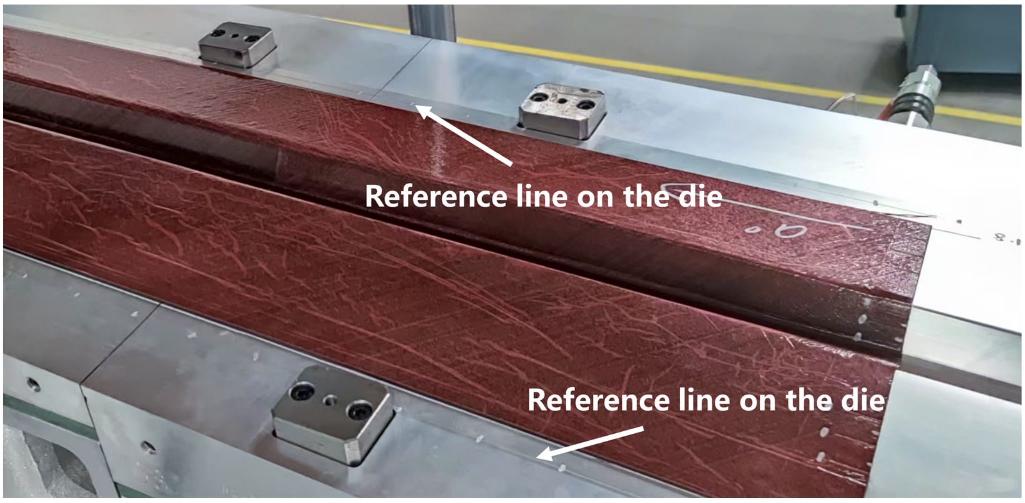

Figure S3 The schematic diagram of reference lines on the die
